# All in One High Quality Genomic DNA and Total RNA Extraction From Nematode Induced Galls for High Throughput Sequencing Purposes

**DOI:** 10.3389/fpls.2019.00657

**Published:** 2019-05-31

**Authors:** Ana Cláudia Silva, Virginia Ruiz-Ferrer, Ángela Martínez-Gómez, Marta Barcala, Carmen Fenoll, Carolina Escobar

**Affiliations:** ^1^Facultad de Ciencias Ambientales y Bioquímica, Universidad de Castilla-La Mancha, Toledo, Spain; ^2^International Research Organization for Advanced Science and Technology (IROAST), Kumamoto University, Kumamoto, Japan

**Keywords:** *Arabidopsis thaliana*, genomic DNA, *Meloidogyne javanica*, nucleic acids integrity, simultaneous RNA/DNA extraction, total RNA

## Abstract

*Meloidogyne* spp. are plant-parasitic nematodes that form a very complex pseudo-organ, called gall, which contains the giant cells (GCs) to nourish them. During the last decade, several groups have been studying the molecular processes accompanying the formation of these structures, combining both transcriptomics and cellular biology. Among others, it was confirmed that a generalized gene repression is a hallmark of early developing GCs formed by *Meloidogyne javanica* in Arabidopsis and tomato. One of the main mechanisms behind this gene repression involve small RNAs (sRNAs) directed gene silencing. This is supported not only by the described action of several microRNAs differentially expressed in galls, but by the differential abundance of 24-nucleotide sRNAs in early developing Arabidopsis galls, particularly those rasiRNAs which are mostly involved in RNA-directed DNA methylation. Their accumulation strongly correlates to the repression of several retrotransposons at pericentromeric regions of Arabidopsis chromosomes in early galls. However, the contribution of this global gene repression to GCs/galls formation and maintenance is still not fully understood. Further detailed studies, as the correlation between gene expression profiles and the methylation state of the chromatin in galls are essential to raise testable working hypotheses. A high quality of isolated DNA and RNA is a requirement to obtain non-biased and comprehensive results. Frequently, the isolation of DNA and RNA is performed from different samples of the same type of biological material. However, subtle differences on epigenetic processes are frequent even among independent biological replicates of the same tissue and may not correlate to those changes on the mRNA population obtained from different biological replicates. Herein, we describe a method that allows the simultaneous extraction and purification of genomic DNA and total RNA from the same biological sample adapted to our biological system. The quality of both nucleic acids from Arabidopsis galls formed by *M. javanica* was high and adequate to construct RNA and DNA libraries for high throughput sequencing used for transcriptomic and epigenetic studies, such as the analysis of the methylation state of the genomic DNA in galls (MethylC-seq) and RNA sequencing (RNAseq). The protocol presents guidance on the described procedure, key notes and troubleshooting.

## Introduction

Plant sedentary endoparasitic nematodes from *Meloidogyne* spp. genus (root-knot nematodes), represent a serious threat to the agricultural production (McCarter, 2009). These obligate parasites are attracted by their hosts and after penetration and migration, they establish within the vascular cylinder forming a pseudo-organ, called gall, that include the giant cells (GCs) used for feeding (Escobar et al., 2015). Both molecular and cell biology studies have contributed to a better understanding of the modifications occurring in galls and GCs, where a generalized gene repression takes place in an early-developing stage (Jammes et al., 2005; Barcala et al., 2010; Portillo et al., 2013; Cabrera et al., 2016). However, the mechanisms that contribute to this gene silencing in early developing GCs are still not clear (Cabrera et al., 2018; Siddique and Grundler, 2018). The involvement of several microRNA-mediated gene silencing of particular gene targets, such as *TCP4, ARF3, TOE1*, and *MYB33* has been recently reported during the root-knot nematode interaction (Zhao et al., 2015; Cabrera et al., 2016; Medina et al., 2017; Díaz-Manzano et al., 2018). Additionally, the accumulation of rasiRNAs (repeat associated small interfering RNAs; Medina et al., 2018; Ruiz-Ferrer et al., 2018) strongly correlates to the repression of several retrotransposons at pericentromeric regions of Arabidopsis chromosomes in early galls (Ruiz-Ferrer et al., 2018). However, mechanisms mediated by epigenetic processes such as RNA-directed DNA methylation (RdDM) in this system are still poorly understood. Deep transcriptomic and methylome analysis will be crucial for a detailed information of those putative mechanisms. For this reason, it is essential to obtain RNA and DNA with high integrity and reproducibility among extractions of independent replicates. Moreover, the possibility to simultaneously extract RNA and DNA from the same biological sample constitutes a great advantage for a suitable correlation analysis between transcriptomic and epigenetic changes in the DNA. Furthermore, it minimizes enormously sample collection, which is a tedious and time consuming procedure due to the small size of galls at early infection stages.

Transcriptome analysis, genome sequencing and bisulphite sequencing are examples of a broad list of molecular studies that are performed routinely nowadays. So far, several protocols of simultaneous purification of DNA and RNA have been published on many research fields as, for example, human tissue and blood (Evans et al., 1998; Radpour et al., 2009), cell culture (Vorreiter et al., 2016), fish embryos (Triant and Whitehead, 2009), microbes (Mcllroy et al., 2008; Hill et al., 2015) or infections by viral pathogens in humans (He et al., 2017). However, there is still scarce information about simultaneous extraction of DNA and RNA on the plant field (Xiong et al., 2011; Oliveira et al., 2015; Hazarika and Singh, 2018). Nevertheless, this procedure remains ill-defined for the structures and tissues formed during the plant-pathogens interaction, and particularly for the nematode-induced galls.

Here, we present a protocol that allows a simultaneous extraction and purification of genomic DNA and total RNA from the same plant tissue sample, focused on feeding structures formed by a plant-parasitic nematode, *Meloidogyne javanica*, in Arabidopsis, and its respective control roots.

## Protocol Overview

In order to obtain galls induced by *M. javanica* and their respective equivalent control root segments from Arabidopsis, Columbia-0 (Col-0), plants are infected with *M. javanica* juveniles, samples are hand dissected, collected and quickly frozen in liquid nitrogen. Tissue disruption is performed in a mortar and pestle with a buffer containing 2-mercaptoethanol and homogenized using a QIAshredder^®^ spin column (QIAGEN^®^). After the homogenization procedure, the lysate is transferred to an AllPrep^®^ DNA Mini spin column (QIAGEN^®^) and centrifuged. We proceed with the RNA purification after a chloroform extraction followed by an incubation with proteinase K. The total RNA is then bound to an RNeasy^®^ Mini spin column (QIAGEN^®^), washed and incubated with DNase. It is finally washed several times and eluted. The genomic DNA extraction is then initiated using the remaining AllPrep^®^ DNA Mini spin column (QIAGEN^®^). The DNA is incubated with proteinase K and RNase (RNase A, QIAGEN^®^, Hilden, Germany), washed and eluted. The genomic DNA is finally concentrated using a vacuum concentrator. Quantification and analysis of the RNA and DNA integrity is then performed using a spectrophotometer (NanoPhotometer^®^ Classic, Implen, Munich, Germany), an electropherogram and a gel electrophoresis.

### Description of Plant Material and Collection

**FIGURE 1:**
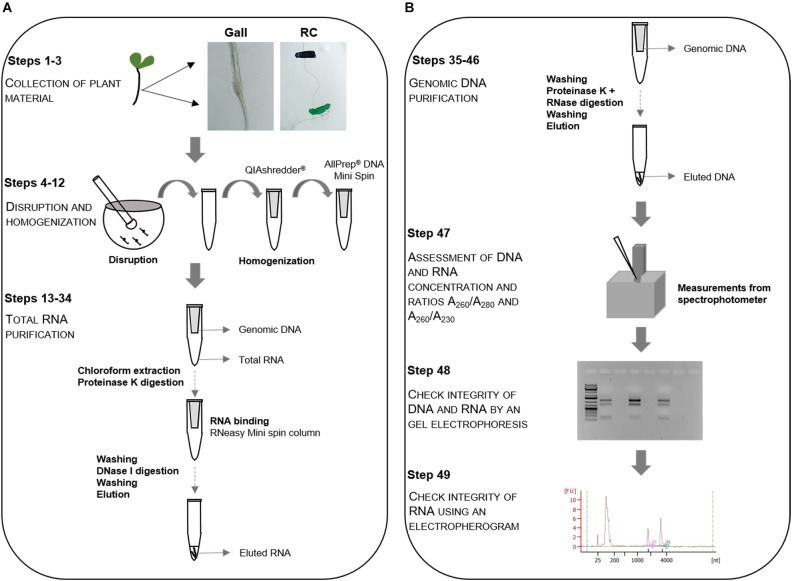
Flowchart of the RNA/DNA extraction procedure. Schematic flowchart for the simultaneous extraction of genomic DNA and total RNA from galls induced by *Meloidogyne javanica* and its respective control root regments (RC). **(A)** Plant material is collected, disrupted in a mortar and pestle and homogenized using a QIAshredder^®^ spin column followed by the total RNA purification. The primary root segments of uninfected plants in equivalent positions to that of the galls in infected plants are considered as control root tissue (steps 1–3). **(B)** RNA purification is followed by purification of the genomic DNA (steps 4–46). Assessment of DNA and RNA purity, quality and integrity is performed in steps 47–49.

Nematode populations are maintained *in vitro* under sterile conditions as described in Díaz-Manzano et al. (2016). *Arabidopsis thaliana* (L.) Heynh. Col-0 seeds are surface sterilized by soaking in 30% commercial bleach with 0.1% v/v Triton^®^ X-100, for 12 min, sown (at a density of 10 seeds per dish) in 90 mm Petri dishes containing Gamborg B5 medium (Gamborg et al., 1968) supplemented with 15 g L^-1^ sucrose and 0.6% Daishin Agar (Duchefa Biochemie, Haarlem, Netherlands), pH 7.0, and kept in the dark at 4°C for 48 h for stratification. Plants are then placed in a growth chamber at 23°C, 30% relative humidity and a long-day photoperiod (16h/8h; light:dark) where they germinate and grow for 5 days vertically. Roots are then inoculated with 10–15 freshly hatched *M. javanica* second stage juveniles (J2). Infections are checked every 24 h under a Leica Mz125 (Leica Microsystems, Switzerland) stereomicroscope in order to establish a penetration and infection timeline (described in Portillo et al., 2013). During the first 48 h of infection monitoring, the plates are maintained horizontally in the dark and then placed again vertically and covered with a gauze to avoid excess of light (Olmo et al., 2017). Galls and uninfected root segments are collected as described in Figure 1, according to Portillo et al. (2013). At 3 days post infection (dpi), 300 galls and 1000 control root segments (RCs) were used for each biological independent replicate. For late time points (14 dpi), we collected 250–300 galls and 500 RCs per replicate. This protocol focuses on the extraction and purification of DNA and RNA from root tissues and galls in *A. thaliana* from *in vitro* cultures. It could potentially be used for the interaction of Arabidopsis with other root pathogens, symbionts or other parasites, yet, specific changes will probably be necessary to adapt it to other plant species or tissues.

### Optimization of the Starting Material

Before the nucleic acid’s extraction from any plant tissue it is important to optimize the amount of starting plant material which might influence the time, concentrations, volumes, column size and temperature used in all steps. Therefore, it is important to perform an initial experiment to determine the minimum starting material needed. Herein, we have tested different amounts of starting material as well as different tissue disruption methods, TissueLyser II from QIAGEN^®^ or mortar and pestle (data not shown). In our hands, the best amount of starting material for a good quality RNA and DNA, accomplishing the requirements for RNA sequencing (RNAseq) and Whole Genome Bisulphite Sequencing analysis (WGBS), was 300 galls and 1000 RCs per biological replicate at 3 dpi and 250 galls and 500 RCs at 14 dpi. The best extraction method was using a mortar and a pestle as shown in Portillo et al. (2006). Taking into consideration the difficulty in collecting the material, we have also measured the weight of a single RC of approximately 1 cm length (Figure 1, Steps 1–3; Collection of plant material) from Arabidopsis in order to have a reference (0.06 mg). 150 galls and 500 RC at 3 dpi are enough to obtain around 5.8 and 5.7 μg of total RNA from galls and RC, respectively, and 1.1 and 1.3 μg of genomic DNA from galls and RC, respectively. This amount of total RNA is suitable to perform some expression analysis as quantitative-real-time-PCR (qRT-PCR; Supplementary Figure S1), yet we do not recommend using this protocol from less than 100 galls and 300 RC as RNA and DNA yield is variable and DNA is hardly detected from small samples. However, in order to have enough DNA and RNA for downstream high throughput procedures and later validation, we recommend at least 1000 pieces of RCs and 300 hand dissected 3 dpi galls.

### Disruption and Homogenization

RNA and DNA are purified from galls and RCs using the AllPrep^®^ DNA/RNA/miRNA Universal Kit from QIAGEN^®^ GmbH (Hilden, Germany) customized protocol (as detailed in AllPrep^®^ DNA/RNA/miRNA Universal Handbook) with several adaptations. These include the disruption, homogenization, RNase A-treated DNA and elution; as this protocol is originally optimized for animal tissue and human whole blood, but not for plant tissue.

Complete disruption of galls and roots are performed using a mortar and pestle. The mortar and pestle are firstly cooled down using liquid nitrogen. When the liquid nitrogen is almost evaporated, the galls or root segments from uninfected plants are placed on the mortar using a sterile spatula and the tissue is grinded thoroughly using the pestle. 300 μL of RLT Plus Buffer (QIAGEN^®^) supplemented with 2-mercaptoethanol (10 μL per mL of RLT Plus Buffer) is added to a liquid nitrogen-frozen mortar with the biological sample and grinded until forming an homogenous paste. When thaw, the lysate is transferred to a 2 mL microcentrifuge tube and the tissue left on the mortar walls is recovered with 50 μL of the same buffer. The lysate is pipetted directly into a QIAshredder^®^ spin column (QIAGEN^®^) and centrifuged for 2 min at 18000 × *g* (centrifugal force). The supernatant is transferred, without disturbing the pellet, to an AllPrep^®^ DNA Mini spin column (QIAGEN^®^) and centrifuged for 30 s at 18000 × *g*. RNA extraction (from the flow-through) is performed immediately and the column containing the DNA is kept in the fridge at 4°C (Figure 1, Steps 4–12; Disruption and homogenization).

### RNA Extraction Procedure

For RNA extraction, the customized protocol proposed by the manufacturer is followed using the upper aqueous phase obtained, after adding 90 μL of chloroform to the flow-through of the AllPrep^®^ DNA Mini spin column (QIAGEN^®^; see former section). A 10 min incubation with 50 μL Proteinase K (QIAGEN^®^) and 100% ethanol is performed before transferring the samples to an RNeasy Mini spin column (QIAGEN^®^). The column is washed with 500 μL of RPE Buffer (QIAGEN^®^) and a 15 min on-column DNase digestion (10 μL DNase I stock solution, included in the kit) is performed. The column is then washed with several solutions including Buffer FRN (QIAGEN^®^), Buffer RPE (QIAGEN^®^) and ethanol, following manufacturer’s instructions. To elute the RNA, 30 μL RNase-free water is added directly to the spin column membrane and centrifuged for 1 min at 9000 × *g* and this step is repeated at least a second time using a new microcentrifuge tube (Figure 1, Steps 13–34; Total RNA purification).

### DNA Extraction Procedure

The spin column kept at 4°C is washed with Buffer AW1 (QIAGEN^®^) and incubated with a mix of 20 μL Proteinase K (QIAGEN^®^) and 0.6 μL RNase A (100 mg/mL, QIAGEN^®^) in Buffer AW1 (QIAGEN^®^) for 5 min at room temperature. Consecutively, washes with Buffer AW1 and AW2, following manufacturer’s instructions, are carried out and the genomic DNA is eluted by adding 100 μL of elution buffer. This step is repeated twice. The three tubes containing 100 μL of eluted DNA each are placed in a vacuum concentrator at room temperature at 40–60 kPa for 6 h, until the volume is reduced approximately to 30 μL. The three eluates are pooled in the same tube if the concentration of the first elution is not sufficient for the experiment (Figure 1, Steps 35–46; Genomic DNA purification).

### Concentration and Quality of Genomic DNA and Total RNA

**FIGURE 2:**
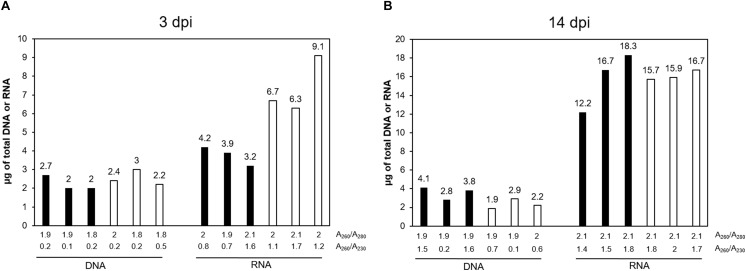
DNA and RNA extraction efficiency for gall and control roots. **(A)** At 3 days post infection (dpi), **(B)** at 14 dpi. *Y*-values represent the amount of total genomic DNA and total RNA, obtained from all eluates. Below the graphic, the A_260_/A_280_ (upper line) and A_260_/A_230_ (lower line) ratios are indicated for each sample (when the eluates were not pooled together, the ratios represented are from the first eluate). From left to right – Black bars represent the total amount of genomic DNA or RNA, as indicated, from 300 galls at 3 dpi to 250 galls at 14 dpi. White bars represent the total amount of genomic DNA or RNA, as indicated, extracted from 1000 segments of control uninfected root tissue equivalent to galls at 3 dpi and 500 equivalent to galls at 14 dpi.

**FIGURE 3:**
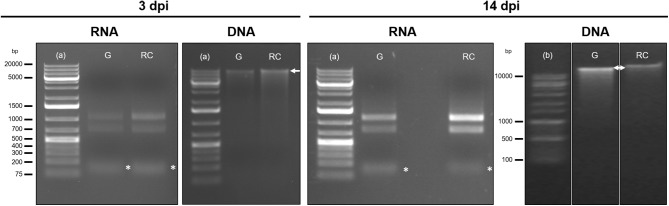
Integrity assessment by gel electrophoresis of total RNA and genomic DNA extracted from galls at 3 days post infection (dpi) and 14 dpi and uninfected control root samples. From the left to the right: samples loaded were RNA and DNA from galls (G) and control roots (RC) samples either at 3 dpi or at 14 dpi, as indicated. A high molecular weight genomic DNA band (>10000 bp) is indicated by a white arrow. Two ribosomal bands are distinguished in the RNA samples and a low molecular weight band, corresponding to the small RNAs represented by an asterisk below the 200 bp marker band (∗). 1% Agarose gels were stained with 6% ethidium bromide; run at 70 V for 40 min for DNA and 30 min for RNA. The gel of DNA samples at 14 dpi was run at 100 V for 30 min. (a) Thermo Scientific^TM^ GeneRuler^TM^ 1 kb Plus DNA Ladder; (b) 1 kb (+) DNA Ladder; bp, base pairs.

Concentration and purity of genomic DNA and total RNA is assessed in a spectrophotometer (NanoPhotometer Classic^®^, Implen, Munich, Germany), using a 3 μL aliquot of the total solutions (Figure 1, Step 47; Assess DNA and RNA concentration and ratios). DNA and RNA purity is estimated from the A_260_/A_280_ and A_260_/A_230_ ratios obtained (Figure 2) and by gel electrophoresis of 100–150 ng of RNA and 50–100 ng of DNA (Figure 3).

DNA integrity is checked through electrophoresis (Figure 1, Step 48; Integrity of RNA and DNA by gel electrophoresis), using an agarose 1% gel. A high molecular weight size band and a very light smear below the bands is indicative of high genomic DNA integrity.

**FIGURE 4:**
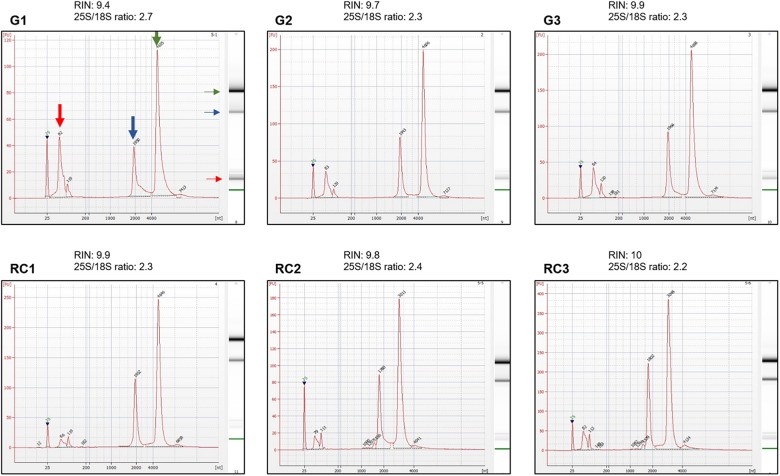
Integrity assessment of RNA samples using electropherograms. Electropherograms of RNA samples for three independent biological replicates of galls, G1–G3, and control roots, RC1–RC3, at 14 days post infection (dpi). Standard size ladder in nucleotides (nt) is shown at the *X*-axis at the bottom of each graph. FU, fluorescent units at the *Y*-axis. The electrophoresis column generated by the program is located at the right side of each sample graph. Red arrow indicates the peak of small RNAs. The positions of 18 and 25S of the ribosomal RNA are indicated by a blue and green arrow, respectively. The electropherograms were performed by an external company (Macrogen, Inc., South Korea) using an Agilent^®^ 2100 Bioanalyzer, RNA Bioanalyzer Pico 6000 chip (Agilent Technologies, Inc., Santa Clara, CA, United States).

RNA integrity is evaluated with an Agilent 2100 Bioanalyzer using the RNA Bioanalyzer Pico 6000 chip (Agilent Technologies, Inc., Santa Clara, CA, United States). In order to obtain the electropherograms, 1 μL of total RNA solution from each sample is used (Figure 1, Step 49; Integrity of RNA by electropherogram). The ratio of the peak areas (25S/18S), corresponding to the 25 and 18S ribosomal RNA (rRNA), the RNA Integrity Number (RIN) and the presence of peaks representing small size RNA bands, are used to assess RNA integrity (Figure 4).

## Materials and Equipment

### Reagents

(1) 2-mercaptoethanol (Sigma-Aldrich^®^, cat. no. M6250).
(2) 2-Propanol (Isopropanol, EMSURE^®^, Merck, cat. no. 109634).
(3) Acetone (VWR^®^, cat. no. 20066.321).
(4) Agarose D1 Medium EEO (Conda, cat. no. 8019.00).
(5) Agilent^®^ High Sensitivity DNA Kit (Agilent Technologies, cat. no. 5067-4626).
(6) AllPrep^®^ DNA/RNA/miRNA Universal Kit (50 preps, QIAGEN^®^, cat. no. 80224). Includes: Buffer RLT Plus; Buffer RPE; Buffer FRN; Buffer AW1; Buffer AW2; Buffer EB and RNase-free water.
(7) Chloroform (analysis grade, stabilized with ethanol, Scharlau, cat. no. CL02031000).
(8) DNA Gel Loading Dye (6X; Thermo Scientific^TM^, cat. no. R0611).
(9) Ethanol absolute (VWR^®^, cat. no. 20821.330).
(10) Ethidium bromide solution (BioReagent, for molecular biology, 10 mg/mL in H_2_O; Sigma-Aldrich^®^, cat. no. E1510).
(11) GeneRuler 1 kb Plus DNA Ladder (Thermo Scientific^TM^, cat. no. SM1331).
(12) Liquid nitrogen.
(13) Proteinase K [included in the AllPrep^®^ DNA/RNA/miRNA Universal Kit, QIAGEN^®^, 2 mL, >600 mAU/mL, solution, QIAGEN^®^ (cat. no. 19131, when sold separately)].
(14) RNase A (2.5 mL, 100 mg/mL, 7000 units/mL, QIAGEN^®^, cat. no. 19101).
(15) RNase-Free DNase Set [with Buffer RDD, included in the AllPrep^®^ DNA/RNA/miRNA Universal Kit, QIAGEN^®^, 1500 Kunitz units, 50 reactions (cat. no. 79254, when sold separately)].
(16) RNase-free water.
(17) TAE Buffer (Tris-Acetate-EDTA, 50× Solution; Fisher BioReagents, Thermo Scientific^TM^, cat. no. BP1332-1).

### Optional

(18) RNaseZap^TM^ RNase Decontamination Solution (Invitrogen^TM^, cat. no. AM9780).

### Reagent Setup

All buffers (AW1, AW2, FRN, RLT Plus, RPE) and the RNase-Free DNase (including Buffer RDD) should be prepared previously accordingly to the manufacturer’s protocol instructions (available at AllPrep^®^ DNA/RNA/miRNA Universal Handbook, QIAGEN^®^).

### Equipment List

(1) Aerosol filter pipet tips 1–100 μL, 1–200 μL, 100–1000 μL (VWR^®^, cat. no. 732-1103, 732-0541, and 732-0534, respectively).
(2) Agarose gel electrophoresis cell Mini-Sub^®^ Cell GT (Bio-Rad Laboratories, Inc., cat. no. 1664270EDU).
(3) Agilent^®^ 2100 Bioanalyzer instrument (Agilent Technologies, cat. no. G2939BA).
(4) Carbon steel scalpel blade n°11 (B Braun^TM^, cat. no. BB511).
(5) Centrifuge (refrigerated, Hettich^®^ Mikro 220R, cat. no. Z724173EU-1EA).
(6) Electrophoresis power supply Power Pac 300 (Bio-Rad Laboratories, Inc., discontinued).
(7) Eppendorf^®^ tubes 1.5 and 2 mL (Eppendorf^®^ Safe-Lock tubes, cat no. 0030120086 and 0030120094, respectively).
(8) Fume hood.
(9) Microcentrifuge Eppendorf^TM^ 5424 (non-refrigerated, Eppendorf^TM^, cat. no. 022620401).
(10) Mortar 25 mL (JIPO, cat. no. 641331211000).
(11) Pestle 115 mm (JIPO, cat. no. 641331213100).
(12) Pipettes Eppendorf^®^ Research^®^ plus 2–20 μL, 20–200 μL, 100–1000 μL (Eppendorf^®^, cat. no. 3123000039, 3123000055 and 3123000063, respectively).
(13) QIAshredder^®^ columns (50 units, QIAGEN^®^, cat. no. 79654).
(14) Spatulas (210 mm, Bochem^TM^, cat. no. 10420441).
(15) Spectrophotometer (NanoPhotometer^®^ Classic, Implen, discontinued).
(16) Stereomicroscope Leica MZ125 (Leica Microsystems, phased out).
(17) Thermoblock (Thermostat dry block, JP Selecta^TM^, cat. no. 7471200).
(18) Tube racks.
(19) Tweezers Dumoxel^®^ 115 mm (Electron Microscopy Sciences^®^, Dumont, style 5A, cat. no. 72720-D).
(20) Vacuum concentrator: centrifuge (RC 10-09, Thermo Electron Industries, model no. 111767780) coupled to a vacuum pump (R-300, Boeco, cat. no. BOE 8830000).

### Equipment Setup

(1) Mortars and pestles (are previously cleaned with pure bleach overnight, abundantly rinsed with distilled water and then autoclaved): prepare a recipient with liquid nitrogen, which will be used to cool down the mortars and pestles during the extraction.
(2) Non-refrigerated centrifuge: set it at 18000 × *g*.
(3) Refrigerated centrifuge: set it at 4°C, 3 min, 18000 × *g*.
(4) Thermoblock: set it at 70°C and place the Eppendorf^®^ tube with Buffer EB (QIAGEN^®^) with enough volume for the three elution steps from the DNA column, 100 μL for each elution.
(5) Vacuum concentrator: in order to get around 30 μL per tube from the 100 μL elution volume (genomic DNA), place the opened tubes in the centrifuge at 1800 × *g* and the vacuum pump at 40–60 kPa for around 6 h. In any case, it is important to check the remaining volume from time to time.

## Stepwise Procedures

The all in one protocol presented here allows the simultaneous extraction of genomic DNA and total RNA, including small RNAs from plant root tissues. As the major procedures during this protocol are related with the extraction and purification of nucleic acids, including RNA, the use of gloves during all stages is recommended. To avoid the presence of RNases we recommend the use of sterile tubes, a clean benchtop and a solution to clean your benchtop, pipettes and other material such as RNaseZap^TM^ RNase Decontamination Solution, as well as aerosol filter pipet tips, especially for RNA to avoid RNase contaminated aerosols. A fume hood should be used at least in the extractions steps where 2-mercaptoethanol is used. The procedure for the extraction and purification of nucleic acids should not take longer than 3 h. In any case, it always depends on the amount of samples used.

## Stages of the Protocol

The presented protocol is divided in four main stages including: (A) collection of plant material; (B) disruption and homogenization of the plant tissue; (C) total RNA purification; (D) genomic DNA purification; and (E) assessment of DNA and RNA concentration, quality and integrity (Table 1).

**Table 1:** Timing overview of each stage of the protocol.

Stage	Steps	Procedure	Runtime
A	1–3	Collection of plant material	Depending on the quantity of material
B	4–12	Disruption and homogenization	∼7 min per sample
C	13–34	Total RNA purification	∼1 h
D	35–46	Genomic DNA purification	∼25 min
E	47–49	Assessment of DNA and RNA concentration, quality and integrity	∼1 h for the gel electrophoresis N/A for the rest of steps


## Stage A. Collection of Plant Material

**Time**: Depending on the type and quantity of plant material

During this stage, all materials and reagents should be cleaned and sterilized beforehand. Be cautious in order to make sure that all material and reagents are RNase and DNase-free.

(1) Use RNaseZap^TM^ or similar to clean all equipment, including the stereomicroscope and the tube racks.
(2) When the biological material comes from *in vitro* culture, it is recommended to drain the excess of water before handling the biological material.
(3) Hand dissect the galls and the control root segments (1 cm) using a scalpel and transfer them, one by one, to the corresponding Eppendorf^®^ tube immersed in liquid nitrogen using tweezers in order to freeze the tissues immediately.

## Key Notes to Keep in Mind During Stage A

(i) Due to its price, RNaseZap^TM^ can be replaced by a low-cost alternative as acetone.
(ii) This is a laborious stage which can take hours. Keep in mind that the material should be always free of RNases and DNases. For this reason, it is important to clean or substitute the scalpels and tweezers frequently (after handling 40–70 samples).

## Stage B. Disruption and Homogenization of the Plant Tissue

**Time**: approximately 10 min per sample

During the disruption and homogenization, all steps, including the centrifugation, should be performed at room temperature. At this stage, the thermoblock should be set up at 70°C and the refrigerated centrifuge at 4°C. 2-mercaptoethanol must be added to the Buffer RLT Plus (QIAGEN^®^) just before use (10 μL 2-mercaptoethanol per 1 mL Buffer RLT Plus, QIAGEN^®^).

### Disruption

(4) Cold down the mortar and pestle using liquid nitrogen.
(5) When the liquid nitrogen is evaporated, place the sample on the mortar and grind thoroughly using the pestle (a spatula can be used to help placing the tissue on the mortar).
(6) Add 300 μL of Buffer RLT Plus (QIAGEN^®^) to the mortar and grind thoroughly using the pestle in order to get a paste while thawing.
(7) When completely thawed, transfer it to a cooled 2 mL microcentrifuge tube.
(8) Clean the mortar walls with 50 μL of Buffer RLT Plus (QIAGEN^®^) and immediately decant the solution into the same tube as in step 7. This step minimizes the loss of biological material on the mortar walls.
(9) Proceed to the next step.

### Homogenization

(10) Transfer the solution into a QIAshredder^®^ spin column (QIAGEN^®^) fixed in a collection tube and centrifuge for 2 min at 18000 × *g*.
(11) Add the homogenized solution, in the collection tube from the previous step, to an AllPrep^®^ DNA Mini spin column (QIAGEN^®^) and centrifuge for 30 s at 18000 × *g*.
(12) Transfer the flow-through into a new 2 mL microcentrifuge tube for the following RNA purification and keep the AllPrep^®^ DNA Mini spin column (QIAGEN^®^) at 4°C for later DNA purification.

## Key Notes to Keep in Mind During Stage B

(i) A small recipient (for instance, 5 mL microcentrifuge tubes) can be used to cool down the mortar and pestle by pouring the liquid nitrogen on them. If a high volume of liquid nitrogen is added to the mortar at once, the biological sample can be splashed out of the mortar. For this reason, it is also important to add the tissue only when all liquid nitrogen is evaporated. It is also possible to cool the mortar by immersion.
(ii) In the case the mortar is too cold, the buffer might get frozen. Start grinding only when a white paste is present and until is thawed.
(iii) After the homogenization, a pellet might be formed at the bottom of the collection tube. Do not disturb it.

## Stage C. Total RNA Purification

**Time**: approximately 1 h

All the following steps are based on the manufacturer’s protocol provided by QIAGEN GmbH (Hilden, Germany) in its AllPrep^®^ DNA/RNA/miRNA Universal Kit’s Handbook. As proposed by the manufacturer, the initial steps’ volumes were adjusted accordingly to the amount of the starting material.

(13) Add 90 μL chloroform to the flow-through from step 12, coming from the homogenization steps, and vortex. Then, centrifuge it using the refrigerated centrifuge at 4°C for 3 min at 18000 × *g* in order to separate the phases.
(14) Transfer the aqueous phase to a new microcentrifuge tube.
(15) Add 50 μL Proteinase K and mix with a pipette.
(16) Add 200 μL of 100% ethanol and mix gently by repeated tube inversion without centrifuging.
(17) Incubate the sample for 10 min at room temperature.
(18) Add another 400 μL of 100% ethanol and mix gently by repeated tube inversion without centrifuging.
(19) Transfer 700 μl of the sample to a RNeasy Mini spin column (QIAGEN^®^) placed in a 2 mL collection tube and centrifuge for 15 s at 18000 × *g*.
(20) After centrifugation, discard the flow-through.
(21) Repeat the previous step, if necessary, with the remaining sample volume in the same column.
(22) Add 500 μl of Buffer RPE (QIAGEN^®^) to the spin column membrane and centrifuge for 15 s at 18000 × *g*.
(23) After centrifugation, discard the flow-through.
(24) Apply a mix of 10 μl DNase I (QIAGEN^®^) stock solution and 70 μl of Buffer RDD (QIAGEN^®^). Add it directly into the spin column membrane.
(25) Incubate it at room temperature for 15 min.
(26) Add 500 μl of Buffer FRN (QIAGEN^®^) to the spin column membrane and centrifuge for 15 s at 18000 × *g*.
(27) After centrifugation, discard the flow-through (**Important: see note ii) from Key Notes from Stage C**).
(28) Add 500 μl of Buffer RPE (QIAGEN^®^) to the spin column membrane and centrifuge for 15 s at 18000 × *g*.
(29) After centrifugation, discard the flow-through.
(30) Add 500 μl of 100% ethanol to the spin column membrane and centrifuge for 2 min at 18000 × *g*.
(31) In order to better wash the column membrane, place the RNeasy Mini spin column (QIAGEN^®^) in a new 2 mL collection tube after discarding the old one with the flow-through and centrifuge for 2 min at 18000 × *g*.
(32) For the final elution, remove the RNeasy Mini spin column (QIAGEN^®^) from the collection tube and place it in a new RNase-free 1.5 mL microcentrifuge tube.
(33) Add 30 μl of RNase-free water directly to the column membrane and centrifuge for 1 min at 9000 × *g*.
(34) Repeat the last step at least once using another 30 μl RNase-free water and using another microcentrifuge tube for a second/third elution.

## Key Notes to Keep in Mind During Stage C

(i) Be careful in step 14 not to mix the phases. If it occurs, in order to get clean purified RNA, you might centrifuge it again to get a sharper separation of organic and aqueous phases.
(ii) In accordance to the manufacturer’s instructions (QIAGEN^®^), between steps 26 and 28, the flow-through from step 27, which is enriched in small RNAs (sRNAs), can be applied again in the spin column and centrifuged in order to enrich the total RNA samples with sRNAs. Alternatively, the eluate of step 27 could be kept as it will be enriched in sRNAs.
(iii) In the case that the concentration of the first eluate is low, several eluates can be combined together in the same tube. In some instances, the second/third elution can show lower A_260_/A_280_ ratios than the first elution.
(iv) After extraction, the RNA should be kept on ice for further processing or stored at -80°C for later studies in order to preserve its integrity.

## Stage D. Genomic DNA Purification

**Time**: approximately 25 min

As for the RNA purification, all the following steps are based on the manufacturer’s protocol provided by QIAGEN GmbH (Hilden, Germany) in its AllPrep^®^ DNA/RNA/miRNA Universal Kit’s Handbook. In the present protocol for DNA purification, the use of RNase and the optimized elution procedure represent the major changes from the manufacturer’s protocol.

(35) Add 350 μl of Buffer AW1 (QIAGEN^®^) to the AllPrep^®^ DNA Mini spin column (QIAGEN^®^) containing the tissue homogenate from step 12 and centrifuge for 15 s at 18000 × *g*.
(36) After centrifugation, discard the flow-through.
(37) Apply the following mixture to the spin column membrane: 20 μl Proteinase K (QIAGEN^®^) and 0.6 μL RNase (QIAGEN^®^) to 60 μl Buffer AW1 (QIAGEN^®^).
(38) Incubate the spin column with the mixture for 5 min at room temperature.
(39) Add 350 μl of Buffer AW1 (QIAGEN^®^) to the spin column membrane and centrifuge for 15 s at 18000 × *g*.
(40) After centrifugation, discard the flow-through.
(41) Add 500 μl of Buffer AW2 (QIAGEN^®^) to the spin column membrane and centrifuge for 2 min at 18000 × *g*.
(42) After centrifugation, discard the flow-through.
(43) For the final elution, remove the AllPrep^®^ DNA Mini spin column (QIAGEN^®^) from the collection tube and place it in a new 1.5 mL microcentrifuge tube.
(44) Add 100 μl Buffer EB (QIAGEN^®^), preheated to 70°C, directly to the spin column membrane. Incubate at room temperature for 1 min and then centrifuge for 1 min at 9000 × *g*.
(45) Repeat step 44 twice with 100 μl of preheated Buffer EB (QIAGEN^®^) each time and using new microcentrifuge tubes.
(46) Place the samples in a vacuum concentrator at room temperature and at 40–60 kPa for 6 h until the solutions reached approximately 30 μL.

## Key Notes to Keep in Mind During Stage D

(i) The mixture from step 37 should be prepared in a separate microcentrifuge tube, vortexed and centrifuged before being applied to the spin column membrane. In this step, the RNase incubation is performed to avoid RNA contamination.
(ii) It is important that the Buffer EB (QIAGEN^®^), from step 44, is preheated to 70°C before the final elution. It should be maintained in the thermoblock between elutions.
(iii) In the case that the concentration of the first eluate is low, several eluates can be combined together in the same tube and concentrated in a speed vacuum concentrator. In some instances, the second and the third elution can show lower A_260_/A_280_ ratios than the first elution.
(v) After extraction, the DNA should be kept on ice for further processing or stored at -20°C to preserve its integrity.

## Stage E. Assessment of DNA and RNA Concentration, Quality and Integrity

(47) Assess the DNA and RNA concentration and quality using a spectrophotometer (Figure 2).
(48) To check the integrity of the DNA and RNA by electrophoresis, run a 1% agarose gel (in TAE buffer) at 70–90 V for 40 min for DNA and at 70–80 V for 20–30 min for RNA. Load 100–150 ng of RNA and 50–100 ng of DNA from each sample (Figure 3).
(49) Assess RNA integrity of the RNA on electropherograms by checking the 25S/18S rRNA ratio and the RIN (Figure 4).

## Key Notes to Keep in Mind During Stage E

(i) It is important to check either the A_260_/A_280_ and A_260_/A_230_ ratios given by the spectrophotometer. An A_260_/A_280_ ratio of 2.0 is accepted as indicative of highly pure RNA. An A_260_/A_280_ ratio of 1.8 is accepted as indicative of highly pure DNA. Pure nucleic acids usually show an A_260_/A_230_ ratio of around 2–2.2.
(ii) It is accepted that pure RNA in the electropherograms show a RIN higher than 6.5, the closest to 10 the best, and a 25S/18S rRNA ratio close to 2 or higher. Also, peaks of low molecular weights, indicative of degradation products, should not be present, expect for the population of sRNAs.
(iii) Be careful not to run the gel electrophoresis with your RNA samples for a long time as the RNA integrity might be influenced by the high temperatures reached during the electrophoresis.
(iv) The electrophoresis cell and combs should be cleaned previously with a normal soap-based detergent and rinsed with distilled water, in order to eliminate any residues and/or nucleic acids-degrading enzymes.

## Results and Discussion

Here, we present a protocol that allows the simultaneous extraction of DNA and total RNA with high quality and enough yield for high throughput sequencing purposes (Figure 1) from the same biological sample of *M. javanica*-induced galls and its respective control root segments.

Due to the banning of nematicides (Ec No1107/2009, 2009), there is an urgent need to search for new control strategies. The study of the mechanisms governing the plant-nematode interaction could bring some light on tools as, e.g., genes or epigenetic signatures that could be used in a future as biotechnological tools. In this respect, an emerging topic coming from the molecular analyses combining transcriptomics with cell biology, such as laser microdissection of giant cells of different plant species, is that early developing GCs are characterized by widespread, but specific, gene repression that includes many microRNAs (Barcala et al., 2010; Portillo et al., 2013; Cabrera et al., 2016; Medina et al., 2017). However, the gene silencing mechanism/s involved is/are not completely understood. One hypothesis is that this gene repression is partially mediated by sRNAs either directly (by RNA interference) or indirectly by RdDM. Studies to reveal important clues on the mechanisms behind this gene repression should consider holistic approaches combining genome wide analysis and gene expression such as the methylome, RNAseq, sRNAseq, etc., of nematode feeding sites compared to uninfected tissues. Subtle differences on epigenetic processes are frequent among independent biological replicates of the same tissue and they may not correlate with changes on the mRNAs or sRNAs population also obtained from different replicates of the same tissue. Therefore, the method described here allows the simultaneous extraction and purification of genomic DNA and total RNA from the same biological sample (from Figure 1–4).

**FIGURE 5:**
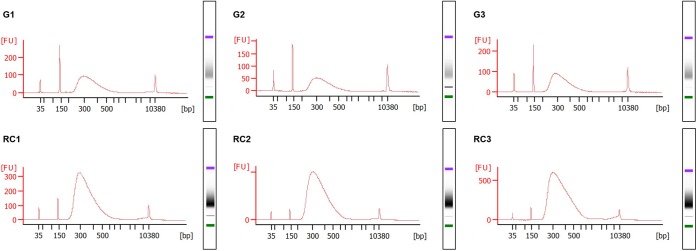
Electropherograms of RNA sequencing (RNA-seq) libraries. TruSeq Stranded mRNA Library Prep Kit from Illumina^®^ was used to prepare the libraries of the 3 days post infection (dpi) samples, strictly following the manufacturer’s instructions. The fragment size distribution of the libraries were checked in the Agilent^®^ 2100 Bioanalyzer using the Agilent^®^ High Sensitivity DNA Kit (Agilent Technologies, Inc., Santa Clara, CA, United States). Gall samples: G1 to G3; Control roots samples: RC1 to RC3. Standard size ladder in nucleotides (bp) is shown at the *X*-axis at the bottom of each graph. FU, fluorescent units at the *Y*-axis. A clear major peak of 300 bp size fragments is observed. The libraries and electropherograms were performed by an external sequencing company (AllGenetics & Biology SL, A Coruña, Spain).

**FIGURE 6:**
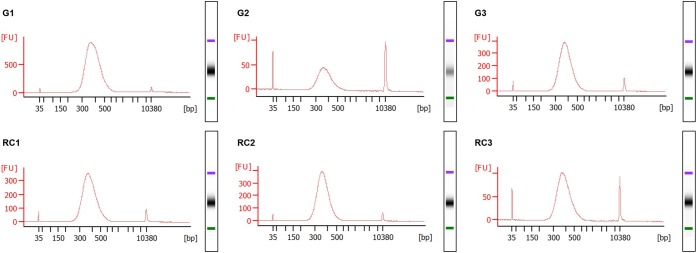
Electropherograms of Whole Genome Bisulfite Sequencing (WGBS) libraries. NEXTflex^®^ Bisulfite-Seq Library Prep Kit (Bioo Scientific) was used to prepare the libraries of the 3 days post infection (dpi) samples, strictly following the manufacturer’s instructions. The fragment size distribution of the libraries was checked in the Agilent^®^ 2100 Bioanalyzer using the Agilent^®^ High Sensitivity DNA Kit (Agilent Technologies, Inc., Santa Clara, CA, United States). Gall samples: G1 to G3; Control roots samples: RC1 to RC3. Standard size ladder in nucleotides (bp) is shown at the *X*-axis at the bottom of each graph. FU, fluorescent units at the *Y*-axis. A clear abundant major peak of around 360 bp size fragments is observed. The libraries and electropherograms were performed by an external sequencing company (AllGenetics & Biology SL, A Coruña, Spain).

We optimized different steps from the manufacturer’s protocol including the amount of the starting material, the volumes used in the disruption, the elution steps and the use of RNase during the DNA purification. For the homogenization, we have also included the QIAshredder^®^ spin column, which is given as an alternative for this step by the manufacturer. DNA and RNA suitable for library construction (Figure 5, 6), for sequencing, and for qRT-PCR analyses (Supplementary Figure S1) and possibly other molecular analysis, were obtained from around 250–300 *M. javanica* galls induced in Arabidopsis roots and 500–1000 control root segments. The DNA and RNA yields ranged from 2 to 2.7 μg; 3.2 to 4.2 μg; respectively for galls at 3 dpi and from 2.8 to 4.1 μg; 12.2 to 18.3 μg; respectively for 14 dpi galls (Figure 2). In all cases A_260_/A_280_ ratios were around 2 for both 3 and 14 dpi samples (Figure 2). However, in some cases, the A_260_/A_230_ ratio obtained was quite low. Some contaminants might contribute to this low ratio, such as salts, carbohydrates or phenol among others. According to the manufacturer of the Kit used, the A_260_/A_230_ ratio of an RNA sample can be reduced even when guanidine thiocyanate, within the extraction buffer, is present at submillimolar concentrations (von Ahlfen and Schlumpberger, 2010). However, the authors claim that in their study, the concentrations of guanidine thiocyanate up to 100 mM in an RNA sample did not compromise the reliability of RT-PCR (von Ahlfen and Schlumpberger, 2010). In our hands, qRT-PCR or libraries for RNAseq were not altered even in the case of this low ratio in the samples used (Supplementary Figure S1 and Figure 5, respectively).

It is important to point that DNA and RNA of good quality could be obtained from lower amount of biological samples as mentioned in the section “optimization of the starting material,” but, in our hands, nucleic acids obtained from lower amount of tissue did not accomplish requirements for high throughput sequencing, particularly, enough DNA yield for the analysis and later validation. Representative electropherograms of 14 dpi galls show the high quality of RNA obtained either from galls or their control tissues (Figure 4) as RIN numbers were never lower than 9.4 and most of them close to 10 in all samples and replicates. In addition, the 25S/18S ratio values ranged from 2.2 in replicate 3 of RC (RC3) to the highest value in replicate 1 of galls (G1; 2.7) indicative of a high RNA integrity. Accordingly, no peaks corresponding to low molecular weight RNAs, indicative of partial degradation, were detected in the electropherograms (Figure 4). However, a major peak was observed between 25 and 200 base pairs (bp) that is likely due to an enrichment of sRNAs in the total RNA (Figure 4). This is in agreement with the gel electrophoresis which shows that the ribosomal RNA was intact with no evident smear and also a prominent band just below the ladder band of 200 bp, presumably corresponding to the sRNAs peak observed in the electropherograms (compare Figure 3 and Figure 4). As explained in the “Key notes to keep in mind during stage C,” an intermediate step can be performed to enrich the sample in small RNAs. However, even if omitted, some of those small RNAs are still present in the sample (Figure 3, 14 dpi; and Figure 4). Additionally, the 3 dpi gall RNA was used to amplify a gene by qRT-PCR, the *CHROMOMETHYLASE 2* (*CMT2*), and its corresponding normalizer, *GLYCERALDEHYDE-3-PHOSPHATE DEHYDROGENASE C2* (*GAPC2*). Clear bands of 60 and 86 bp for *CMT2* and *GAPC2*, respectively, were observed (Supplementary Figure S1) and reproducible in all 3 technical replicates used, indicating that the RNA obtained is suitable for qRT-PCR analysis.

In respect to the DNA quality, the gel electrophoresis indicated that a high molecular weight band was present in all samples either at 3 dpi or 14 dpi (Figure 3). The incubation with RNase during the genomic DNA purification (Stage D) allowed us to obtain RNA-free genomic DNA as bands of low molecular weight were not detected (Figure 3).

After construction of the RNA and DNA libraries for RNAseq and WGBS, electropherograms show a fragment size distribution with a major peak around 300 bp in the RNA libraries and around 360 bp in the DNA libraries (Figure 5, 6) indicative of good quality libraries for subsequent RNAseq and WGBS, respectively. Additionally, the genomic DNA extracted and purified with this method was also appropriate to use in an ELISA based Kit (MethylFlash^TM^ Global DNA Methylation (5-mC) ELISA Easy Kit (Colorimetric), EpiGentek) to measure the global DNA methylation state of galls and control roots. Both independent biological and technical replicates gave reproducible results (data not shown).

Although, the use of this protocol has led us to reliable results, during the course of the protocol optimization, we have encountered several problems (see Table 2 for troubleshooting). The main limiting problem was the amount of starting material. For that, as mentioned above, we weighted the material and optimized the minimum amount needed for the extraction. When the amount of starting material was low, the quantity and quality of the DNA and RNA was affected, which could lead to deficient library construction. During the optimization, we have also used different disruption methods as a TissueLyser II (QIAGEN^®^) and a mortar and pestle. The second option, in addition of being cheaper and consequently available in all laboratories, resulted more efficient (data not shown), as mentioned also by Portillo et al. (2006). Therefore, with the changes introduced, this protocol could be also helpful to obtain high quality nucleic acids from other Arabidopsis tissues, and could be easily extended to other structures produced after the infection of other plant-parasitic nematodes, as well as other plant-pathogens.

**Table 2:** Troubleshooting: list of recommendations to overcome any artifacts or pitfalls during the whole procedure.

Problem	Step (s)	Possible reason	Recommendation (s)
Fail to pour the sample in the mortar	5	Tissues are already thawed; Tissues are too thin and stick to the tubes	Keep the sample tube frozen until pouring; Use a sterile spatula to help
Low total RNA yield	13–34	Insufficient disruption; Low starting material; RNA is still bound in the column	Use the pestle with concentric movements vigorously; Try to weight the material and increase the starting material following our recommendations described in the protocol; Incubate the column a few minutes with the elution solution and centrifuge
Low genomic DNA yield	35–46	The same as described for “low total RNA yield”	The same as described for “low total RNA yield”
DNA contaminated with RNA	37–38	Insufficient incubation with RNase	Extend incubation time or RNase concentration
Degraded RNA	1–34	Frozen samples not well preserved at -80°C; Handling material in an inappropriate way; RNase contamination of the biological samples	Before disruption, keep your samples always frozen; Handle carefully your samples using during all procedure RNase-free and sterilized material and wear gloves; Use a different pipette set and tips when extracting DNA or RNA; Keep RNA on ice for further processing or stored at -80°C for later studies
Low A_260_/A_230_ ratio	47	Contamination with guanidine thiocyanate	During the washes and elutions, try to reduce the contact of the buffers and solution with the spin column walls
Cannot visualize bands in the gel electrophoresis	48	Low concentration of DNA and/or RNA	Increase the concentration of DNA and/or RNA loaded in the gel


Available methods to extract and purify nucleic acids from plants are numerous, yet, this is the first time that a simultaneous extraction and purification of both genomic DNA and total RNA from the same biological sample of galls and plant roots of high quality and suitable for current high throughput sequencing methods, is described. The importance of this protocol relies on the fact that obtaining RNA and DNA of high quality from the same biological sample is a great advantage for studies combining gene expression and epigenetics. Therefore, it is particularly helpful, to study changes in the genomic DNA leading to changes in gene expression as the cells of the tissues used for RNA and DNA extraction are exactly the same as well as their cellular status.

## Conclusion

The method herein described was successfully adapted for the simultaneous extraction of nucleic acids, genomic DNA and total RNA, from the same biological sample. Here we describe the case of the *M. javanica* galls formed in Arabidopsis roots as compared to control root segments. Understanding the mechanisms leading to changes in gene expression during the formation and maintenance of the feeding sites induced by these pathogens, nowadays entangle combined studies at the transcriptomic and epigenomic level. In this context, the importance of this protocol relies on the fact that obtaining RNA and DNA of high quality from the same biological sample is a great advantage for the study of epigenetic alterations in the DNA, related to changes in gene expression within the same tissue. One of the main reasons is that the cellular status of the tissue used for RNA and DNA extraction is exactly the same as they are obtained simultaneously. Therefore, we believe that this method can be also extended for the simultaneous extraction of high quality RNA and DNA from other Arabidopsis tissues or other structures induced by other plant-parasitic nematodes or even possibly other pathogens/symbionts interactions.

## Data Availability

All datasets generated for this study are included in the manuscript and/or the Supplementary Files.

## Author Contributions

AS, VR-F, ÁM-G, and MB performed most of the experiments related with the protocol. AS and CE aimed the protocol. VR-F, MB, and CE guided AS for the experiments. AS and CE wrote the manuscript. The final version was supervised by AS, VR-F, CF, and CE. All authors read the manuscript.

## Conflict of Interest Statement

The authors declare that the research was conducted in the absence of any commercial or financial relationships that could be construed as a potential conflict of interest.

## Supplementary Material

FIGURE S1 Agarose gel electrophoresis of quantitative-real-time-PCR (qRT- PCR) products of the *CHROMOMETHYLASE 2* gene (*CMT2*). qRT-PCR products of *CMT2* (AT4G19020) and *GAPC2* (*GLYCERALDEHYDE-3-PHOSPHATE DEHYDROGENASE C2*; AT1G13440), used frequently as a normalizer for qRT-PCR. 12.5 ng of the initial total RNA from galls was used in each of the three technical replicates (1–3) for the two tested genes. The corresponding cDNAs were amplified with the primers in Supplementary Table S1 and single clean amplicon bands (60 bp in the case of *CMT2* and 86 bp in the case of *GAPC2*) can be observed. Ethidium bromide-stained agarose gel electrophoresis. NTC, No template control; Ladder, Thermo Scientific^TM^ GeneRuler^TM^ 1 kb Plus DNA Ladder; bp, base pairs.

TABLE S1 List of primers used for qRT-PCR.
