# Supplementary figures and images for: All in One High Quality Genomic DNA and Total RNA Extraction From Nematode Induced Galls for High Throughput Sequencing Purposes
Source: Front Plant Sci. 2019 May 31;10:657. doi: 10.3389/fpls.2019.00657 (PMC6554733; doi:10.3389/fpls.2019.00657)

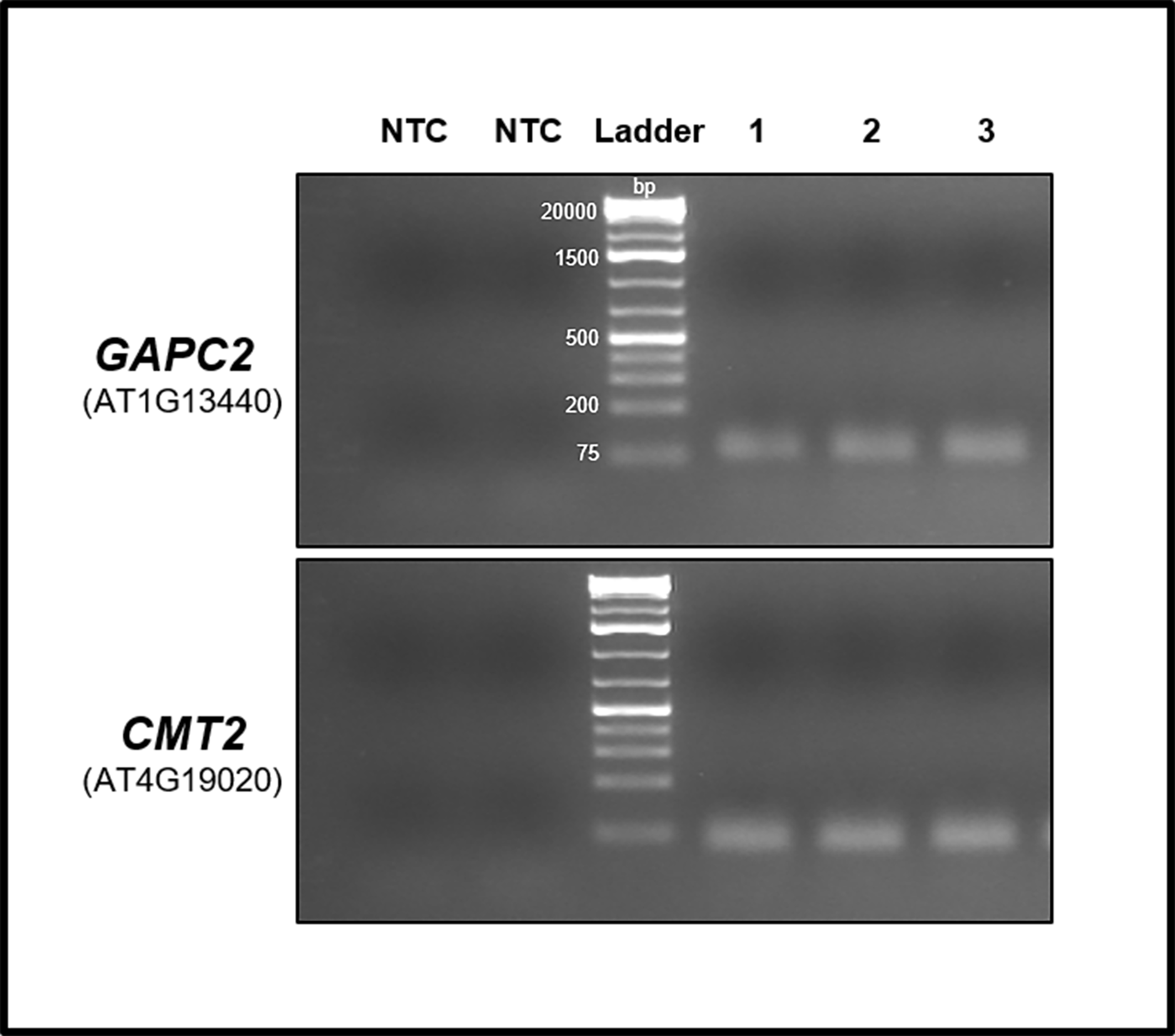

Supplement: FIGURE S1 — Agarose gel electrophoresis of quantitative-real-time-PCR (qRT- PCR) products of the CHROMOMETHYLASE 2 gene (CMT2). qRT-PCR products of CMT2 (AT4G19020) and GAPC2 (GLYCERALDEHYDE-3-PHOSPHATE DEHYDROGENASE C2; AT1G13440), used frequently as a normalizer for qRT-PCR. 12.5 ng of the initial total RNA from galls was used in each of the three technical replicates (1–3) for the two tested genes. The corresponding cDNAs were amplified with the primers in Supplementary Table S1 and single clean amplicon bands (60 bp in the case of CMT2 and 86 bp in the case of GAPC2) can be observed. Ethidium bromide-stained agarose gel electrophoresis. NTC, No template control; Ladder, Thermo ScientificTM GeneRulerTM 1 kb Plus DNA Ladder; bp, base pairs. [file Image_1.TIF]
