# Supplementary material for: All in One High Quality Genomic DNA and Total RNA Extraction From Nematode Induced Galls for High Throughput Sequencing Purposes
Source: Front Plant Sci. 2019 May 31;10:657. doi: 10.3389/fpls.2019.00657 (PMC6554733; doi:10.3389/fpls.2019.00657)
Supplement: TABLE S1 — List of primers used for qRT-PCR. [file Table_1.DOCX]

| **Primer** | **Gene name** | **Gene ID** | **Sequence (5’-3’)** | **Amplicon size (bp)** |
| --- | --- | --- | --- | --- |
| CMT2_F | *CMT2* | AT4G19020 | GGTTACCAAAGGGGGAGTTT | 60 |
| CMT2_R | *CMT2* | AT4G19020 | AAGCAGCGGCTGTGACTTAC | 60 |
| GAPC2_F | *GAPC2* | AT1G13440 | TGTGGTTGAGTTCGTACTGTTCTGA | 86 |
| GAPC2_R | *GAPC2* | AT1G13440 | CTGCGCATGGAATCAGTGAA | 86 |
